# Implementation of grip strength measurement in medicine for older people wards as part of routine admission assessment: identifying facilitators and barriers using a theory-led intervention

**DOI:** 10.1186/s12877-018-0768-5

**Published:** 2018-03-22

**Authors:** Kinda Ibrahim, Carl R. May, Harnish P. Patel, Mark Baxter, Avan A. Sayer, Helen C. Roberts

**Affiliations:** 1 Academic Geriatric Medicine, University of Southampton, Southampton General Hospital, Mailpoint 807, Tremona Road, Southampton, SO16 6YD UK; 2 Faculty of Health Sciences, University of Southampton, Highfield, Southampton, SO17 1BJ UK; 3 NIHR CLAHRC: Wessex, University of Southampton, Highfield, Southampton, SO17 1BJ UK; 4 Medicine for Older People, Southampton General Hospital, Mailpoint 63, Tremona Road, Southampton, SO16 6YD UK; 5 AGE Research Group, Institute of Neuroscience, Faculty of Medical Sciences, Newcastle University, Newcastle, UK; 6 NIHR Newcastle Biomedical Research Centre, Newcastle upon Tyne Hospitals NHS Foundation Trust and Newcastle University, Newcastle, UK

**Keywords:** Older, Inpatients, Grip strength, Implementation, Clinical practice, Hospital

## Abstract

**Background:**

Low grip strength in older inpatients is associated with poor healthcare outcomes including longer length of stay and mortality. Measuring grip strength is simple and inexpensive. However, it is not routinely used in clinical practice. We aimed to evaluate the implementation of grip strength measurement into routine clinical practice.

**Methods:**

This implementation study was a mixed methods study based in five acute medical wards for older people in one UK hospital. Intervention design and implementation evaluation were based on Normalization Process Theory (NPT). A training program was developed and delivered to enable staff to measure grip strength and use a care plan for patients with low grip strength. Routine implementation and monitoring was assessed using the “implementation outcome variables” proposed by WHO: adoption, coverage, acceptability, fidelity, and costs analysis. Enablers and barriers of implementation were identified.

**Results:**

One hundred fifty-five nursing staff were trained, 63% in just 3 weeks. Adoption and monthly coverage of grip strength measurement varied between 25 and 80% patients across wards. 81% of female patients and 75% of male patients assessed had low grip strength (< 27 kg for men and < 16 kg for women). Staff and patients found grip measurement easy, cheap and potentially beneficial in identifying high-risk patients. The total cost of implementation across five wards over 12 months was less than £2302. Using NPT, interviews identified enablers and barriers. Enablers included: highly motivated ward champions, managerial support, engagement strategies, shared commitment, and integration into staff and ward daily routines. Barriers included lack of managerial and staff support, and high turnover of staff, managers and champions.

**Conclusions:**

Training a large number of nurses to routinely implement grip strength measurement of older patients was feasible, acceptable and inexpensive. Champions’ motivation, managerial support, and shared staff commitment were important for the uptake and normalisation of grip strength measurement. A high percentage of older patients were identified to be at risk of poor healthcare outcomes and would benefit from nutritional and exercise interventions. Measuring grip strength in these patients could provide an opportunity to identify those with normal grip strength for fast tracking through admission to discharge thereby reducing length of stay.

**Trial registration:**

Clinicaltrials.gov NCTO2447445. Registered May 18, 2015.

**Electronic supplementary material:**

The online version of this article (10.1186/s12877-018-0768-5) contains supplementary material, which is available to authorized users.

## Background

People over 65 years old make up nearly two thirds (65%) of people admitted to hospital in the UK [1, 2]. Many of these patients have multiple medical problems including frailty and sarcopenia. Frailty is defined as “a decline in multiple body systems, which increases an individual’s vulnerability to changes in health or their environment” [3]. The prevalence of frailty among older patients in hospitals reaches 25–40% [4, 5]. Sarcopenia, the loss of skeletal muscle mass and strength with age, is an ageing syndrome [6] with a prevalence in hospitalised older people of around 10%, and 30% among nursing home residents [7].

Grip strength is central to both frailty and sarcopenia [6, 8]. Poor healthcare outcomes including increased risk of falls [9], morbidity [10], death [11], longer length of hospital stay and higher hospitalization costs [12–14] are all associated with low grip strength among older patients. The European Working Group on Sarcopenia in Older people (EWGSOP) recommend screening for sarcopenia among all people aged over 65 years by measuring first gait speed and then grip strength [15]. However, some authors suggest that using grip strength alone can be enough to screen for frailty and sarcopenia [16] and is especially useful for people who have difficulty walking. Progressive resistance training with nutritional intervention has been reported to be feasible, safe and effective for improving muscle strength and treating frailty and sarcopenia [17–21].

Routinely measuring grip strength could be useful to identify older inpatients at higher risk of poor healthcare outcomes potentially allowing the implementation of appropriate interventions. Despite the evidence of its validity, reliability, and simplicity and the wealth of research evidence of the value of assessing grip strength in clinical practice and its links to poor health outcomes and, the measurement of grip strength is currently limited to research studies and not used in routine practice [22].

The implementation of evidence-based practice in busy and understaffed healthcare organisations like the National Health Service (NHS) can be challenging [23]. To implement new practices, effective implementation strategies and individual and organisational barriers must be recognised [24]. Interactive training and educational outreach visits, whereby trainers visit staff where they practice and provide them with information to change how they practice, were reported to improve the delivered care for patients and could have modest effects in changing health professional practice [25, 26]. There is recent evidence that Normalisation Process Theory (NPT) can provide a theoretical framework that identifies, characterises and explains the mechanisms that motivate and shape implementation processes in a busy healthcare setting [27, 28], and which focuses attention on the work that professionals and patients need to do to translate knowledge into routine clinical practice. Therefore, the aim of this study was to assess the feasibility and acceptability of implementing grip strength measurement into routine clinical practice in the UK. We attempted to simultaneously address the following:

1. The feasibility of training staff on measuring grip strength of older inpatients.
2. The adoption, coverage, fidelity and cost of implementation
3. The enablers and barriers of implementation

## Methods

### Study design

The study design was based on Normalization Process Theory (NPT) which focusses on evaluating change in practice and examining the adoption and integration of interventions into organizational routines [29]. The NPT four constructs: coherence or sense-making, cognitive participation, collective action, and reflexive monitoring formed the basis for the implementation process in this study. The detailed protocol for this study is published [30].

This was a mixed methods theory-led study. It consisted of (a) intervention design and delivery informed by NPT; (b) implementation evaluation using qualitative methods; routinely collected patient data; and analysis of implementations costs. Full ethical approval was obtained from NRES Committee South West – Frenchay (REC REFERENCE 15/SW/2012). This study was reported following the Standards for Reporting Implementation Studies (StaRI) [31].

### Settings and participants

The study was conducted in five acute medical wards for older people (three female wards, two male) in one hospital in England. All patients admitted to the study wards, including those who have dementia or cognitive impairment, were eligible to perform the grip strength test. Patients receiving end-of-life care were excluded from the test.

### Implementation strategy

#### Staff training and education

##### Development of the training programme and care plan

The main implementation strategy that was deemed to be relevant and useful for integrating grip strength routinely in daily practice was interactive educational training. The training programme was developed to match the four constructs of NPT to allow staff to make sense of the new practice (implementing grip strength measurement routinely) and to permit cognitive participation from staff in interactive and fun training activities. It included an educational leaflet, and a practical demonstration of grip strength measurement (role play) using a Jamar dynamometer (Patterson medical Ltd) according to a standardised protocol [32]. Grip strength was tested with patients seated. Two measurements with each hand were used instead of three measurements since our recent research with acute medical inpatients suggests that the third attempt is tiring and is rarely the maximum value. A brief break of approximately 1 min was allowed between each measurement and the maximum value was recorded in kilograms (Kg). Patients who were unable to sit out on a chair had their grip strength measured sitting up in bed.

The educational leaflet provided information about grip strength, the clinical relevance of low grip strength values, how to measure patient’s grip strength and use the care plan for patients with low grip strength (< 27 kg for men and < 16 kg for women) (see Additional file 1). The care plan directed that patients with low grip strength should receive dietary review of need for oral nutritional supplements, and review of patient’s mobility by a physiotherapist to consider prescribing resistance exercises. The care plan included space to record the reasons if grip strength could not be measured e.g. inability to understand instructions. Patients who were unable to complete the grip measurement were considered high risk.

##### Delivery of the training

Training was delivered daily for three consecutive weeks on the wards by the first author (KI). Additional training sessions were arranged during the study for staff who could not attend during this period and new staff. Training was incorporated into regular educational sessions and induction programmes and occurred in group or one-to-one sessions as appropriate. Staff measured the grip strength of a colleague to develop the necessary skills and to demonstrate their competency. The number, grade, and ward base of staff attending training sessions was recorded. Staff evaluated the training sessions by answering nine simple questions using a five–point rating scale. They were able to discuss any issues related to measuring grip strength so that these could be addressed before starting implementation.

#### Other implementation strategies

The implementation strategies used, in addition to education and training, included: administrative support through establishing a study steering group, regular monitoring and feedback, and incorporation into documentation process (see Table 1).

**Table 1:** Implementation strategies used to enable adoption and integration of grip strength measurement

Implementation strategies	activities
Education and training	Deliver training to nursing staff, doctors, and therapists.
Administrative support	Establishing a clinical steering group including nursing staff from each ward, a dietician lead, a physiotherapist, and the lead of the department education team. Regular meetings every 2–3 months to discuss progress, on-going training needs in each ward, address any identified potential barriers, share successful stories and good experiences and guide the implementation process. Meeting minutes were emailed to ward managers and senior nurses.
Concurrent monitoring and feedback	Regular review of patients’ records to assess coverage was communicated to the clinical staff (either in person or via emails) to promote the implementation process and to inform ongoing change efforts. The department newsletters were also used to facilitate the implementation process to promote training, disseminate any update or progress, acknowledge success (at individual and ward levels), and encourage implementation. The employee of the month was awarded several times to individuals who encouraged the use of grip strength measurement in their wards.
Incorporation into documentation process	The grip strength care plan was added to patient’s routine admission booklet.

### Implementation intervention and monitoring

Each study ward received a Jamar dynamometer (five Jamars in total) and copies of the care plan. The dynamometers were calibrated at the beginning and end of the study and their measurement accuracy against known weights was checked every 3 months during the study period. The implementation intervention involved measuring grip strength of all eligible patients within 3 days of admission twice in each hand using a standardised protocol and recording the maximum value in kilograms (Kg). Then the care plan (referring the patient to therapist and prescribing ONS) should be followed for those with low grip strength.

Grip strength implementation was monitored and evaluated by assessing the relevant “implementation outcome variables” proposed by the World Health Organisation (WHO): adoption (the intention to try the service), coverage (the degree to which those with the greatest need received the service), fidelity (the extent to which the service is implemented as prescribed in the original protocol), acceptability (the extent to which the service is agreeable), and costs (total cost of service in context).

To monitor and assess implementation a number of methods was used including:

1. Observation

During the implementation period, the first author (KI) visited the study wards on a weekly basis to promote the adoption of routine grip strength measurement. Field notes included observations on training sessions, formal and informal meetings/discussions with ward managers and other staff as well as the all implementation activities. [33].

2) Collection and analysis of routinely collected patient data

#### Monitoring of grip strength measurement

Patients’ clinical records (nursing and medical notes) on each study ward were reviewed every 1–2 weeks to collect data on 1) the number/ percentage of eligible patients on the ward who had their grip strength measured and the range of values obtained, 2) the number/ percentage of patients who had low grip strength values, 3) the number/ percentage of patients with low grip strength who had received the care plan interventions. As staff were trained to measure grip strength within three days of admission, patients who had a length of stay less than three days at the time of the audit were excluded from the audit data.

#### Monitoring of care plan use

Use of the care plan for low grip strength was indicated by two label stickers (one to refer the patient to therapy team and one to ask the medical team to prescribe Oral Nutritional Supplements ONS) placed in patients’ medical notes. In order to examine whether patients actually received the relevant interventions, the records of a random sample of 86 patients across the study wards were reviewed. This sample included patients who had low grip strength who had at least one sticker in their medical notes. Information was extracted on whether and when nutritional supplements and exercises were given to patients.

3) Semi-structured interviews/focus groups (assess adoption and acceptability)

Qualitative data was collected to assess the acceptability of grip strength measurement to both staff and patients, and to identify the enablers and barriers to its routine use in clinical practice. Eight interviews and three focus groups (two - three participants in each group) were conducted with a purposive sample of 15 staff member (seven nursing staff including four ward champions, two consultant geriatricians, four therapists and two dieticians). The staff interview schedules were semi-structured and used NPT constructs to understand how and what changes were taken by staff to adopt routine implementation of grip strength, how and whether the grip strength care plan was actioned, and whether the routine implementation was acceptable (See Additional file 2). Staff interviews took place on the wards towards the end of the implementation period to capture the actual experience of staff and any changes in practice related to grip strength implementation. A purposive sample of eight patients (five men and three women with high and low grip strength) across the study wards was recruited. Patient interviews were conducted at the patient’s bedside within three days of grip strength measurement to maximise recall. Patients were asked about their experience and knowledge of the purpose of grip strength measurement, their views on the experience, and whether they would do it again. Written consent was obtained from each participant and interviews/focus groups were audio-recorded and transcribed verbatim.

### Data management and analysis

#### Quantitative data

Data were double entered into database and each participant was assigned a unique identification number***.*** Descriptive statistics using the statistical software package IBM SPSS statistics 22 was used to summarising the main quantitative results. Descriptive data was summarised using mean and standard deviation (SD), median and inter-quartile range (IQR) and/or number (percent) as appropriate for the type of data (continuous, normally distributed or not, categorical). The feasibility of training staff was described including staff numbers, discipline, grade, and ward, with comparison across the five study wards. Descriptive statistics were used to describe the coverage data abstracted from the clinical records and compare the practice and implementation of grip strength measurement across the different wards.

#### Qualitative data

Data analysis took place in two phases to avoid forcing the data into categories predetermined by the theoretical framework. An initial thematic analysis followed Brown and Clark [34] steps: familiarisation with the data, coding, searching for themes, reviewing themes, defining and naming themes, and writing up, was conducted by KI and HR and underwent a number of iterations. A descriptive coding scheme was developed from transcripts and based on participants’ perceptions and experiences. Coding proceeded until no more new data developed from analysis (i.e data saturation). In the second phase of analysis, we mapped emergent data themes to the NPT framework checking for fit. A software program for analysing qualitative data (e.g, NVivo 10) was used to facilitate data analysis. The findings of the study were enhanced by the use of different methods and the emphasis on purposive sampling.

#### Costs of implementation

The implementation costs were recorded as the cost of equipment, deliverer of staff training and provisional costs. The cost of training was calculated based on the grades of staff delivering and attending the training. Implementation provisional cost per patient was also recorded.

## Results

### Feasibility and acceptability of training staff on measuring grip strength

**Fig. 1:**
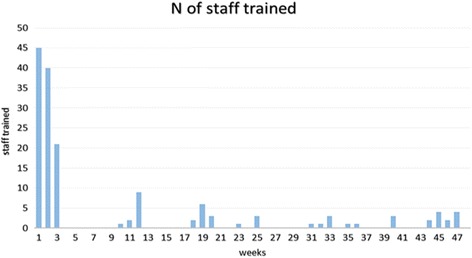
The number of staff trained over one year

The training sessions integrated well within the daily routine of staff and caused minimum disruption to their ward activities. Typically, 1–3 sessions occurred daily during the first 3 weeks lasting around 20 min. In total, 155 / 176 (88%) nursing staff were trained. 98 (63%) staff were trained in 36 sessions during the initial 3 weeks (Fig. 1) and 40 staff were trained in an additional 24 sessions during the study period. Additionally, grip strength training was incorporated into two induction days for 17 new staff. Across the study wards 85 (55%) nursing staff (bands 5, 6, & 7), 45 (29%) healthcare assistants HCAs (band 2–3), 15 (10%) associate practitioners (band 4), and 10 (6%) students were trained. Additional seminars (*n* = 3) were held to educate medical and therapy staff.

All trained nursing staff were recorded to be competent in measuring grip strength. All trained staff found the educational leaflet and the practical session helpful. 99% felt confident in measuring patients’ grip strength and 97% had confidence in using the care plan. 85% thought that they were likely to measure grip strength and 80% thought grip strength would be integrated in their daily practice. Only 4% felt that they needed more training.

### Adoption and coverage of routine grip strength measurement

**Fig. 2:**
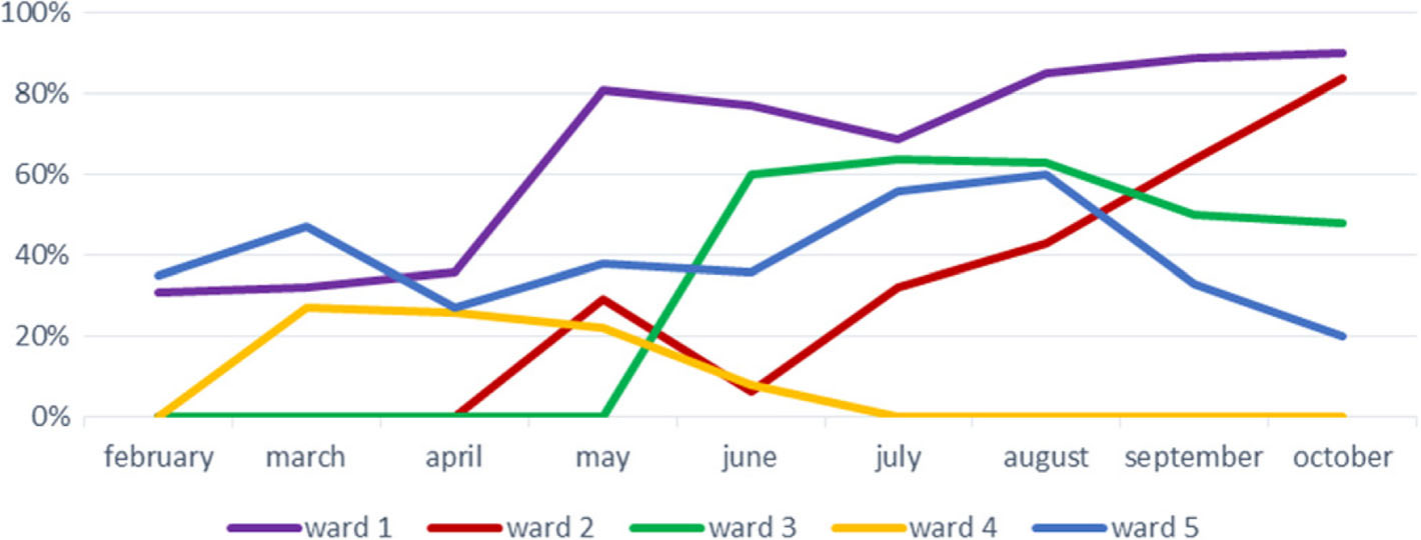
Coverage of grip strength across the study wards over 9 months period

Adoption and coverage of grip strength measurement varied across the study wards (see Fig. 2). One key outcome of the steering group meeting was to nominate ward champion(s) to promote adoption, encourage wider staff engagement, develop strategies to enhance adoption, report training needs, and link with the research team. Regular visits from the research team to the study wards, formal and informal discussions with the ward staff and managers, and observing staff performing the test with their patients were important factors in facilitating the initial adoption of grip strength measurement.

Adoption and coverage of grip strength measurement varied between 0% and 100% across the study wards reflecting different practices. For example, a supportive ward manager and a keen ward champion who valued the new test facilitated adoption on ward 1. Weekly coverage in this ward ranged between 65% and 100% (average 80%). By comparison, coverage in ward 5 ranged between 0% and 93% (average 40%). This variability reflected a lack of shared commitment by other staff. Grip strength measurement was performed mainly by the ward champion and 0% coverage reflected the periods when the ward champion was off work. In ward 4 constant changes in ward managers and lack of support from other staff members were barriers to continue the implementation.

Ward managers in wards 2 &3 initially perceived grip strength measurement as unsuitable for older inpatients due to either inability to sit up or lacking cognitive capacity to perform the test. They were reluctant to add an extra job to nursing staff and perceived the test to be the therapist’s role. However, these concerns were addressed by the research team through constant meetings, visits, and education. The support of the ward manager and keen ward champions was again key to successful implementation and the average weekly coverage was 58% and 70% in these wards.

### Intervention Fidelity

The regular review of patients’ clinical records during the implementation period (9 months) identified 2043 eligible patients, of whom 811 patients had their grip strength measured, reflecting the variation in adoption between wards. The care plans were fully completed as prescribed in the original protocol revealing high fidelity. 655/811 (81%) had performed the grip test of whom 472 (72%) were female and 183 (28%) were male. 81% of female patients had low grip strength (median 11 kg) and 74.5% of male patients (median 20 kg). 156 (19%) were unable to do the test therefore considered at high-risk and the care plan was completed. Documented reasons for inability to measure grip strength included: confusion 28 (18%), severe dementia 31 (20%), inability to understand English / instructions 29 (19%), patient refused 31 (20%), unwell patients who are unable to squeeze 27 (17%), aggressive patients 7 (4%), and patients with severe arthritis 3 (2%).

However, the fidelity of activating of the grip strength care plan varied across the study wards. The care plan was activated (placing the ONS and physiotherapy stickers in patients’ medical records) among almost all patients who were identified to be at high risk in wards 3&5, whereas more variation was observed in the remaining wards. Review of patients’ medical records and electronic prescribing (*n* = 86) revealed that among those who had the ONS sticker 60% were prescribed ONS. In comparison, 20% of those who had the physiotherapy stickers were offered exercises (bed or chair exercises mainly). Only 13% of patients with low grip strength (who had both stickers) were offered exercises and ONS.

### Acceptability of grip strength measurement

#### Patients’ acceptability

Most patients found the measurement easy and straightforward and none felt that it was painful or had concerns about it (See Table 2). Yet, there was recognition from three participants that it could be hard. All patients expressed readiness to repeat the test again if they were asked to. They found the timing of the test was convenient to them -three recalled the test took place in the morning. Seven patients reported that they completed the test while they were sitting on a chair.

**Table 2:** Acceptability of routine grip strength implementation

Acceptability	Quotes
Patient’s acceptability	I mean it was so harmless that you didn’t mind doing it…Quite happy to do it because let’s face it it’ll be, it’s ultimately for my own good isn’t it? (Patient 1, Male, Grip Strength = 26) Yeah that was fairly, you know, clear. You had to sort of keep it steady and then squeeze the first one hand and then change over and you did the other one. And then I think we did it again to just to make sure, cos, you know, possibly the same reading, yeah (Patient 3, Female, Grip strength = 35) I think it could be important to judge that but I don’t know if there is any other means of judging muscle strengths, because you don’t know what it was before do you? So how, how can you tell from just one result. I mean if it’s sort of adds to information which helps I think that’s a good thing (Patient 4, Male, Grip strength = 34) Just had to squeeze my hand on the machine sort of testing, sort of you know muscle strength that kind of thing (Patient 5, Male, Grip strength = 18) It was quite stiff I thought, you had to really press hard to…You just try your best and I’ve got to squeeze as hard as I can (Patient 6, Female, Grip strength = 20) Well it provides long term information to help find out how strong people are normally and if they can manage on their own then it seems a good idea (Patient 8, Male, Grip strength = 20)
Staff acceptability	I think, well if you reach the goal, or in order for the doctors to prescribe Fortisips or supplements and to be more aware that the patient doesn’t have much strength, I think yes it is because it doesn’t take us long so, if it helps the patient then I am happy to do it of course (Nurse 6) I think it’s as good, I think the key is that we highlight these people so we can try and manage them appropriately, and certainly everything I’ve read about grip strength which seems to be positive and supportive that it’s picking up the right people (Consultant 2) I think it’s good, it could be useful. I think it’s a quick thing to do. Obviously it’s just as a general rule, highlighting somebody might be a risk of frailty then; with such a quick test I think it is good… it’s positive. ………but it’s not quite embedded into our sort of daily practice as a unit, not just as a therapy team (Therapist 1) I think there was an element sometimes I kind of get ‘oh phew they have been prescribed’. Sometimes it’s great, it’s probably beneficial. But it’s just that kind of small matter of the overlap on. Cos I do think there is a service, I do think if there is a place that, I’ve always said that, but it’s kind of making sure that you’re not overlapping on work (Dietician 1)

All patients felt that there was a rationale for using the grip strength test as part of routine assessment of older patients. Different reasons were reported by patients including: accepting the science and research behind the test, information obtained could assist health professionals provide patients with the necessary help, and it was seen as a good method to identify weakness and predict whether people can manage on their own. However, two patients questioned the correlation between arm strength and leg strength and asked how only one result can judge prognosis or changes in health.

#### Staff acceptability

The majority of staff had positive views about using grip strength measurement in routine practice. It was seen a cheap and quick test that could identify older patients who have weak muscle strength for further management. Consultants and therapists discussed the potential evidence of the grip test in picking up the right people who might be frail and at risk of poor healthcare outcomes compared to other available frailty tools which have poor validity. Dieticians perceived that routine grip strength measurement could be beneficial by allowing early prescription of necessary supplements to older people. Nevertheless, they had some reservation about the possible overlap with their work when oral nutritional supplements were suggested to unsuitable patients.

### Costs of implementation

The total cost of routine implementation of grip strength across five wards over 12 months was estimated between £2218 (Band 5) and £2302 (Band 7) according to the seniority of staff members who delivered the training. The mean provisional cost of grip strength measurement per patient (time required to perform the grip strength test by a member of staff) was £5.78 and ranged between £4 (Band 2–3) and £10 (Band 7) according to the seniority of staff.

### Enablers and barriers for implementation of routine grip strength measurement

Thematic findings and supporting quotes from the interviews and focus groups illustrating participants’ experiences with the grip strength implementation are presented with the NPT constructs in Tables 3, 4, 5, and 6.

**Table 3:** Coherence-supporting quotes

Coherence	Supporting quotes
Staff awareness	I think when we start, when I talk doing the grip test I used to be asked ‘what is this?’ and now I check with the Sister and she knows they need to do. It was difficult, I think because it was one more thing we need to do, Now is really easy nowadays, (Nurse 3) Make sure that when we tested grip strength on them they got the advised plan in terms of oral nutritional supplements and to highlight patients who haven’t had their grip strength tested to the ward staff (Consultant 2) We did talk about it a few times because we have a once a week team meeting, so went through it a couple of times in there and said ‘look if any patients’ been identified as low grip, we need to be making sure we give them an exercise programme, provided they’re appropriate’ (Therapist 4)
Staff coherence and rational	I have noticed it could speed up things in doing it, because if you get an obese woman, and you think, okay she’s not going to need supplements because she’s overweight, but she’s still weak what you going to do? that’s when I see the logic, (Nurse 4) I think, in terms of grip strength study, what it has done for, certainly us, me and my team, is make us more aware of trying to pick those people up and invest some time in it that we probably weren’t doing before (Consultant 1)
Patient’s comprehension	I have only had a couple of patients that have refused, but that’s because of their dementia, not because they don’t want to do it. cos they just don’t understand or, had a stroke and can’t use their arms, that but generally most people will do it (Nurse 1) Just had to squeeze my hand on the machine sort of testing, sort of you know muscle strength that kind of thing (Patient 5, Male, Grip strength = 18)
Test and interventions specification	Yes, once we know how to do it I don’t think there, it is easy yeah. It doesn’t take long You just do it and straightaway if they are at high risk, we just document on the medical notes and put the sticker (Nurse 6) It’s quite easy to do I would say, especially when you’ve got the sheets there already made up for you. All you need to do is prescribe how many, number wise, that you want them to do but then most of them that are cognitively fine, will do it (Therapist 2)

**Table 4:** Cognitive participation-supporting quotes

Cognitive participation	Supporting quotes
Buy in	All of us just received the training and our manager told us now we have to do all of it as a part of an admission. And that’s it. We just integrate it as part of admission (Nurse 6) I think it’s not changed our day to day caseload sort or anything, because I think it was agreed that patients wouldn’t be sent to physio purely on the basis of grip strength (Therapist 1)
Champions fit and motivation	I just wanted to do it for the ward, really, and make a difference. I’m not one to moan so I’ll just do, follow orders. (Nurse 4) Motivation was getting involved in new scheme with just like, hoping it will get going and everyone would do it (Nurse 7)
Engagement strategies	So sometimes ideally I fill out all the papers, and then I just say ‘here we go do the grip strengths today’ (Nurse 1) When we have the admission, there’s one of the papers that we put straightaway in the folders. Even if they are admitted overnight we put in ready in the folders in order for the day staff will be able to complete it (Nurse 6)

**Table 5:** Collective action-supporting quotes

Collective action	Supporting quotes
Shared commitment	I just didn’t get the backing from, the, well the staff really cos it just wasn’t getting done which is such a shame cos I tried and tried (Nurse 7) I’m well happy about my team because they want to do the things and they trust me about this situation (Nurse 3).
Integration	And after lunch they’ve eaten and maybe we reposition them first and then do the grip,... I just do it as I’ve fitted it in to my routine so I’d always wait until after lunch and do it (Nurse 4) if we have a patient admitted during the day, as long as we are doing her admission paper, property check list or anything else, that’s another check that we have to do is the grip test (Nurse 6)
Activation of care plan	I think, it’s to me it’s quite clear. It’s a sticker stuck in your notes and I tend to read all the notes that have gone on before (yeah) it’s quite transparent so when they ring it and say their grip strength is low, (Consultant 1) We aren’t picking up patients who are just on, were just on the grip strength, because we have enough patients on our radar, as is anyway. (Therapist 4) There could be a conversation I’ve already had with the patient who doesn’t want to go that way. They want to do food first and that’s what we’re kind of aiming for, and then all of a sudden, you know, you kind of feel you’re being slightly undermined cos there’s another sticker (Dietician 1)

**Table 6:** Reflexive monitoring-supporting quotes

Reflexive monitoring	Supporting quotes
Performance appraisal	It’s because I constantly look at the board because that’s part of my job and I see grip strength stickers up there, think gotta do that. (Nurse 1) I mean, we’re pretty good as a team at looking at the white boards, if somebody’s has got a, um, one of the fists on the whiteboards I’ll go in them and sort of make sure they’ve got their exercise programme (Therapist 4) There was one time I put a sticker in, and the same time the doctor wanted the folder and then she looked at it and said ah I’d better prescribe some Fortisips for this patient’….. And there was a physio, he was a Band 7 and said that we’re the only ward consistently doing it. It makes me feel happy (Nurse 4).
Monitoring results	I’m not so much happy about the results because all my patient is like high risk….. Sometimes I tell. You need to ‘don’t worry but exercise’ and eat some more healthy, because we need to make you more strong’ (Nurse 3) Sometimes we saw the patient is really strong, he’s eighty years, but he is really strong but in the tests…He scored low. so I am surprised about this, yes, and sometimes the family are surprised too because ‘oh really only this’? (Nurse 6) I had a woman yesterday, I thought she won’t do more than 10, but she was doing like 24, 25 (Nurse 5) Occasionally you are surprised by people, particularly men that they do better than you imagine they’re going to do. It’s interesting.(Consultant 1) I’ve seen it in the notes and things, but a lot of my patients, I would say like 85% have got the little grip strength (Therapist 2)
Normalisation	it’s like your routine and doing all we really need to do, we know this is important for patient, important for family, important for us, they start to do it (Nurse 3) I don’t know why it’s, I don’t know why people act any differently towards that, than they would the weighing chair, I don’t know why they’re treating that any different, (Nurse 4) Yes. I think all of us now; we know that’s other job that we have to do yeah (Nurse 6) I think, and they’ve sort of maintained that they still do the grip. They’ve sort of, they’ve got it as part of, it’s all integrated into part of their working practice, I’m not sure if all wards have done that, you know what I mean. That’s what I see, but not all wards are quite there yet (Consultant 1)

#### Coherence: For whom and how does grip strength measurement and its care plan make sense?

Making sense of grip strength measurement varied among health professionals and over time and was an important factor to facilitate implementation. Some nursing staff were unaware of grip strength at the beginning but with time many started to make sense of the relevance of the test and developed the initiative to perform it (see Table 3). Measuring patients’ grip strength allowed some nursing staff and ward champions to see the rationale behind performing the test, especially when patients had unexpected results such as overweight patients with low grip strength. Yet, some were still uncertain about the benefits of measuring grip strength because of inability to see the end results. Consultants were aware of and positive about the routine use of grip strength measurement. One consultant took the initiative to teach her medical teams about the research and the importance and relevance of the grip test. Consultants explained how grip strength measurement made them realise the need to identify patients who are at risk of sarcopenia and other poor healthcare outcomes and invest more time with them. Senior therapists were aware of the grip strength measurement, whereas junior therapists were not at the beginning. However, junior therapists mentioned how implementing grip strength measurement made them appreciate the value of giving exercises to more patients. They reported that staff need to understand the importance of giving exercises and mobilising patients to help facilitate their discharge.

Patients also made sense of the purpose of grip strength measurement and understood the instructions. Nursing staff reported that it was fairly easy and practical to engage patients who were able to understand the instructions. One HCA reported that he treated all patients equally including those with cognitive dysfunction in terms of instructions given. Nursing staff found the grip strength test and its care plan and related stickers easy to complete. Therapists reported that the exercises given to patients were straightforward and easy to follow by older patients if they were cognitively well.

#### Cognitive participation: Support and engagement with implementing the grip strength measurement

Managerial support was recognised by nursing staff as an important factor that facilitated adoption and implementation of routine grip strength measurement (see Table 4). Some nursing staff discussed how grip strength was integrated in their admission assessments as a result of support from their ward managers. Other staff reported that implementation in their ward could have improved if there was more support from their managers. Despite staff coherence, there was lack of buy-in from therapy team who reported that patients would not be seen by physios purely on basis of grip strength results. This was due to lack of staff and facilities.

A key component of the implementation was selecting keen and enthusiastic ward champion(s) to help adopt and roll-out the new measurement. Ward managers selected the champions and their seniority varied from ward sister, nurse staff, healthcare assistants, to a student nurse. The motivation rather than seniority of ward champion appeared to influence the success of implementation. One ward (ward 2) displayed a high turnover of ward champions causing fluctuating in coverage and implementation. This was due to lack of individual motivation and coherence. Three wards, which displayed good coverage, had the same ward champions during the study period (wards 1, 3 & 5). These champions viewed themselves as the right person to take on the champion role. They had different motivations including: the wish to make a difference to their wards, involving in a new scheme, and viewing grip strength a part of their job descriptions.

Champions undertook concerted efforts to engage other staff in the implementation of grip strength measurement. Engagement strategies included adding grip strength to handovers, using environmental clues as reminders (i.e. graphical posters and wall checklist in the bays), preparing the grip strength paperwork and delegating staff to perform the test, and /or adding grip strength to the nursing assessments checklist. Others used night shift staff to check clinical records and create a list of the patients who needed the grip test for the nurse in charge the next day.

#### Collective action: How was grip strength measurement integrated into ward routine admission procedures?

Creating a supportive environment established a shared commitment that facilitated implementing grip strength in some wards (see Table 5). Wider shared commitment from staff was evident in ward 1 with the highest coverage. Other wards had lower levels of shared commitment with only a limited number of staff were keen to measure grip strength. Lack of perceived responsibility and relying on ward champions created less supportive communities in these wards. In the wards (2&3) where there was more than one champion, there was a vibrant commitment shared among the champions themselves, described as “working as a team” or “grip partners”. Lack of managerial support and inability to create a supportive environment led to early termination of grip strength implementation in ward 4.

Integration of grip strength measurement was achieved in most of the study wards. Some ward champions described how they included grip strength measurement in their daily routine. For example one champion (ward 3) described choosing after lunch as the best time to do the test as patients would be already in a sitting position. Grip strength care plans were added to patients’ admission booklets with the routine nursing assessments.

Activation of care plans regarding therapist involvement and prescribing ONS appeared to be challenging on some wards. Consultants described the stickers in patients’ notes as a prompt to prescribe supplements. They also described differences in practice and engagement across the wards. Dieticians found the grip strength measurement a good idea in highlighting high risk patients. However, they expressed some reservation regarding some occasions when ONS stickers were placed in medical notes of unsuitable patients such as those who were Nil By Mouth, had swallowing difficulties, refused supplements, or had lactose intolerance. The therapists appreciated the need to give exercises to patients with low grip strength but in reality they did not feel able to deliver this additional service within their current staffing levels.

#### Reflexive monitoring: How was the implementation of grip strength measurement monitored?

Monitoring and reflecting on performance was an important factor in the implementation process (See Table 6). Participants used a number of ways to monitor their performance. For example, nursing staff and therapists used the grip strength magnets on the boards to evaluate their coverage and identify patients who still needed to be assessed. Ward champions mentioned occasions when they received positive verbal feedback from medical and therapy teams on their performance and witnessed some doctors prescribing ONS as a result of using the stickers. Lack of feedback from therapists on the benefits of exercises was reported as a barrier for using the stickers every time.

In addition, interviewees talked about the results of their patients’ grip strength. There was a general consensus that the majority of patients had low grip strength levels which was consistent with the data collected from regular reviews. One therapist mentioned that almost 85% of the patients were at high risk and it was difficult to manage all these patients within their limited resources. However, nurses and consultants reported that patients’ grip strength were surprising sometimes. This was identified as an advantage of the grip strength test in recognising those patients who were described by one consultant as “middle ground”. Consultants explained how grip strength measurement had added more information about patient’s health and helped them justify and fully understand patient’s current and future health status. Some nurses took the opportunity to educate their patients who had low grip strength about the importance of healthy diet and exercises.

Some nursing staff described a sense of satisfaction with the degree of support they had and the extent to which grip strength measurement was embedded and normalised in their current practice. Some reported that grip strength became part of the routine nursing assessment and stated that every nurse should think about it that way, questioning why some treated it differently to other nursing assessments. Evidence of normalisation included stopping using the label “grip strength study” and referring to the new practice as the grip strength test in all admission paperwork across the whole study wards. However, there was still a significant variation in the extent of normalisation across the study wards, which was reported by consultants and therapists.

A summary description of the differences in implementation practice across the five wards is presented in Table 7.

**Table 7:** Description of the differences in implementation across the five wards

Wards	Factors affecting implementation of grip strength measurement
Ward 1	Highly motivated ward champion (a nurse) Buy-in from the ward manager High shared commitment from other staff Engagement strategies included incorporating grip strength test in handover, adding the care plan to admission booklet, and using the grip strength magnets on the board. The ward champion was awarded the employee of the month award for excellence. Grip strength test was normalised in this ward.
Ward 2	Initially there was high turnover in ward champions and ward managers. Then two nominated keen champions (2 nurses) led the implementation process with the support of a new ward manager. Little shared commitment from other staff
Ward 3	Initially there was lack of buy in form the ward manager and lack of perceived responsibility. Then 2 highly motivated ward champions (Healthcare assistant and Associate practitioner) were nominated to lead implementation. Supportive new ward manager No shared commitment from other staff was achieved. Engagement strategies included using the grip strength magnets on the board to monitor performance, include the grip test in the bays checklist, adding the care plan to the admission booklet, allocating certain time during the day to perform the grip test. The ward champions were awarded the employee of the month award for extraordinary efforts.
Ward 4	Highly motivated ward champion (a healthcare assistant). Engagement strategies included placing visual reminders in each bay. The ward champion was awarded the employee of the month award. Unsupportive ward manager No shared commitment from other staff
Ward 5	Highly motivated ward champion (a senior nurse) Supportive ward manager Low shared commitment from other staff Engagement strategies included using the grip strength magnets on the board, allocating the mission to measure grip strength to other staff

## Discussion

Implementation of routine grip strength measurement among older people on admission to hospital proved to be feasible. It was practical, cheap and fairly easy to train a large number of staff to measure grip strength in a relatively short period of time in a busy clinical environment. The adoption and implementation of grip strength varied across the wards. Highly motivated champions, managerial support and shared commitment by other staff were key factors for successful implementation. Inability to build a collective understanding of and commitment to the new measurement and lack of managerial support resulted in inconsistent implementation in some wards. A high proportion of older patients had low grip strength and would benefit from early interventions focusing on diet and exercise. Measuring grip strength in this group of older patients could also provide the opportunity to identify those with normal grip strength and fast track them through admission to discharge. A study by Puig-Domingo et al. [35], evaluating muscle strength and successful ageing, found it to be a helpful clinical evaluation tool and a Japanese study investigating the optimal physical or cognitive test to screen for falls risk in frail older people found that the most practical physical test was grip strength [36]. It should be noted that grip strength is unlikely to change within an admission in response to hospital interventions but is useful to stratify an inpatient population.

Staff and patients found the grip strength measurement and its care plan easy to complete, and beneficial in identifying patients at risk of poor healthcare outcomes especially those who might be missed with other routine assessments [37]. Activating the care plan interventions, specifically physical exercises, to manage sarcopenia and low muscle strength was challenging. This was mainly due to lack of therapists’ support which reflected the high workload of a limited number of staff.

Training large number of staff for a specific task is well documented both in secondary care [38, 39] and primary care [40, 41]. However, there is very limited evidence in the literature on training staff to measure grip strength in hospital setting. In one study, in-service trained registered nurses measured the grip strength of 213 patients with pneumonia who were admitted to a medical pulmonary unit of an urban teaching hospital [42]. This study did not report on the number of staff who received training or how they were trained. Another study, used two in-service Intensive Care Unit (ICU) physical therapists to measure grip strength on the day of awaking for a sample of 29 patients with critical illness who were mechanically ventilated for more than 48 h [43]. However, this is the first study to report on the feasibility of training a large number of staff to measure grip strength of patients as part of routine clinical practice.

The dedication that ward champions showed was remarkable. The characteristic of the champion rather than their seniority was the main determinant of successful implementation. The champion role was successful among delegated individuals if they were highly motivated to do the job, appreciated the benefits of the new test, and effectively communicated and negotiated with their staff peers and managers [44]. Most champions believed that they were well-equipped to fulfil the role and to facilitate the adoption of the new test. Every ward was encouraged initially to determine how best to fit grip strength measurement into their daily routine. Yet, regular meetings allowed champions to share their stories and experiences facilitating implementing the best practice across the study wards.

There is a strong evidence of the effectiveness of progressive resistance exercises in increasing muscle strength among older people, especially when accompanied with nutritional supplements [45–47]. However, delivering resistance exercises routinely to older inpatients who were identified to have low grip strength was challenging. The main reasons reported by therapists were organisational factors such as limited number of staff and work overload, individual factors such as lack of support from senior therapists, and patient-related factors such as dementia. A study reported that only 27% of patients recalled being encouraged by hospital staff to exercise [48].

### Study strengths and limitations

The study has several strengths. For the first time we have shown that it is feasible to train a large number of nursing staff to implement routine measurement of grip strength among older people admitted to hospital. Using a mixed method study design, we have incorporated both qualitative and quantitative research methods to further validate data. Data triangulation (combining data collected from different resources, using different methods and at different times) and investigator triangulation (use of different observers to minimise individual bias) were used to enhance the quality of the work. Yet, our study has also some limitations. This study was conducted in one university hospital where staff might have positive attitudes towards research and implementation of evidence-based research findings into practice. Therefore, findings of this study may not generalizable to hospitals which might be less active in research or among different patient populations. Additionally, we did not evaluate the proportion of trained staff who performed the grip strength measurement.

### Future research

Research on nursing work in acute care settings has shown that contextual factors, for example knowledge of the unit and routine workflow, influence nursing practice. Therefore, it is important to replicate this work in other departments and hospitals to compare variation in clinical practices. Furthermore, this study included very old patients (80 years old and over), the majority of whom were identified to have low grip strength. Implementing the appropriate intervention (mainly exercises) was found to be challenging at this age group. It would be more helpful to target old inpatients at earlier age (65+) to assess the feasibility of providing the appropriate interventions, mainly exercises. Future research should also focus on evaluating the cost-effectiveness of implementing grip strength in practice.

### Implications for wider implementation

Suggestions that may facilitate implementation of grip strength measurement routinely in busy understaffed clinical practice include:

- Liaising closely with and gaining support from ward managers and team leaders (therapy manager and dietetic leader). Ensuring that they are well briefed about the rationale for and long-term outcomes of measuring grip strength.
- Nominating highly motivated and active champion(s) in each participating ward. Their roles include for example; engaging other staff in the implementation process, identifying training needs, liaising with the nurses and the research team, and developing strategies to facilitate implementation.
- Establishing a steering group to facilitate implementation, disseminate updates of progress, share experience, and identify facilitators and barriers along the implementation process.
- Facilitating ownership of methods to adopt and implement grip strength in each ward.
- Providing ongoing training, integrated in the daily work of staff, and included in the induction of new staff.
- Agreeing on a process for collecting information to monitor performance at individual and ward levels. For example, using magnets on the white board proved to be an effective way for monitoring performance and coverage.

## Conclusions

Successful adoption and implementation of routine grip strength measurement among older patients on admission to medicine for older people wards was variably achieved. The NPT offered a framework for identifying specific factors that enabled implementation, as well as areas to target for future research. This study demonstrated the importance of ward champions’ motivation, managerial support, and staff shared commitment for the uptake and normalisation of routine grip strength measurement. Grip strength measurement identified a high percentage of older patients with weaker muscle strength who might be at risk of poor healthcare outcomes. As a result, routine prescription of ONS and exercises in the study wards was adopted by managers. We propose that measuring grip strength in this group could also provide an opportunity to identify those who have normal grip strength allowing potentially to fast track them through admission to discharge.

## Additional files

Additional file 1: “Care plan for Grip Strength Measurement” that need to be completed by nursing staff within 1–3 days of admission or transfer to the ward. (PDF 341 kb)

Additional file 2: Semi-structured interview/focus group schedule for staff and patients to evaluate the acceptability, facilitators and barriers of routine implementation of grip strength measurement. (DOCX 18 kb)

## Abbreviations

EWGSOP: Working Group on Sarcopenia in Older people
HCA: HealthCare Assistant
ICU: Intensive Care Unit
IQR: Inter-Quartile Range
NHS: National Health Service
NPT: Normalisation Process Theory
ONS: Oral Nutritional Supplements
SD: Standard deviation
WHO: World Health Organisation
