# Supplementary material for: Implementation of grip strength measurement in medicine for older people wards as part of routine admission assessment: identifying facilitators and barriers using a theory-led intervention
Source: BMC Geriatr. 2018 Mar 22;18:79. doi: 10.1186/s12877-018-0768-5 (PMC5865333; doi:10.1186/s12877-018-0768-5)
Supplement: Supplementary file 1 — “Care plan for Grip Strength Measurement” that need to be completed by nursing staff within 1–3 days of admission or transfer to the ward. (PDF 341 kb) [file 12877_2018_768_MOESM1_ESM.pdf]

Care plan for Grip Strength Measurement

To be completed within 1-3 days of admission or transfer to the ward

Patients Details	Admission date	*Date of assessing GS*	*Ward*	*Name of assessor*
Name:<br>Age:<br>Gender:				

**Right hand (kg)**	**Left hand (kg)**
Grip strength measurement NO 1=	Grip strength measurement NO 1=
Grip strength measurement NO 2=	Grip strength measurement NO 2=

Maximum grip strength measurement=

High risk
Maximum Grip less than 27kg for men,
Less than 16 kg for women

Intervention plan

1- Review patient's medical care.
2- Follow the ONS care pathway (see over).Pleas ask medical team to prescribe Fortisips compact b.d
3- Refer the patient to Physio for progressive resistance training.

Lower risk
Maximum Grip is 27 kg or more for men,
16 kg or more for women

Refer for usual clinical care

Communication and documentation

1- Place the grip strength sticker on the care plan.
2- Place the completed the grip strength care plan in the patient's medical note.
3- Place Grip strength magnet on Patient Status at a Glance (PSAG) Board.
4- Record the patients with low grip strength on handover sheet.

Record reasons for not completing the care plan:

Oral Nutrition Support Pathway (as part of the GRImP study)

Please follow the following steps:

- Ask medical team to prescribe Fortisip Compact b.d. (amend flavour and temperature to maximise intake).
- Complete accurate, daily food record charts.
- Offer assistance with eating and drinking as required. Involve Meal Time Assistant unless contraindicated e.g. texture modified diet/thickened fluids.
- Identify patient's food preferences from family/carers and inform ward host if patient is not able to communicate this for themselves.
- Offer Complan soup in place of the soup of the day from the menu.
- Offer appropriate snacks between meals via the diet grid e.g. sandwiches, cheese and biscuits, extra yogurts/puddings.
- Encourage 500ml (1pint) of full cream milk per day in milky drinks, including a bedtime milky drink.
- Weigh weekly and document on MUST paperwork.

Continue this action plan until discharge from hospital.

Refer the patient to dietetic department <u>only</u> if:

- MUST score increases
- NBM or consistently eating <½ meals & not managing Fortisip Compact twice a day
- Weight loss continues/ >15%
- Artificial feeding required
- Re-feeding complications develop eg low phosphate, potassium, magnesium
- Modified texture diet needed, requiring specialist input
- Renal function deteriorating, requiring specialist dietetic advice (e.g. potassium>5.5)
