# Supplementary material for: Implementation of grip strength measurement in medicine for older people wards as part of routine admission assessment: identifying facilitators and barriers using a theory-led intervention
Source: BMC Geriatr. 2018 Mar 22;18:79. doi: 10.1186/s12877-018-0768-5 (PMC5865333; doi:10.1186/s12877-018-0768-5)
Supplement: Supplementary file 2 — Semi-structured interview/focus group schedule for staff and patients to evaluate the acceptability, facilitators and barriers of routine implementation of grip strength measurement. (DOCX 18 kb) [file 12877_2018_768_MOESM2_ESM.docx]

**Semi-structured interview/focus group schedule for staff and patients to evaluate the acceptability, facilitators and barriers of routine implementation of grip strength measurement**

**Semi-structured interview/focus group schedules for nursing staff**

I would like first to ask you little bit about the training session you received few months ago,

1. What did you think of the actual training sessions on measuring GS?
2. To what extent do you think that the training was beneficial or not to you personally?
3. After completing the training, how comfortable were you to start measuring GS of older patients? Did you feel you needed any more training?

Now let’s move to discuss your views and experience with measuring GS,

1. What motivated you to start measuring GS? Were there any shared commitments by the nursing and medical staff in your ward to start using GS measurement?
2. What is your experience with measuring GS? What did you have to do? How did you find using the equipment (Jamar dynamometer)? How did you engage patients and perform the test?
3. How did you report the results? Have you completed any paperwork? How practical was it to do so?
4. Has your experience changed over time? How? Were there any barriers or difficulties in measuring GS for older patients in your ward?
5. How practical was it to complete the care plan actions for those patients with low grip strength? Were there any barriers?
6. How has GS assessment been integrated into the ward routine practice? How did GS assessment fit with other things that you needed to do? How did you negotiate and prioritise your daily tasks?
7. In general, do you think that measuring GS routinely is acceptable? Do you think it is a useful part of your work or not?

**Semi-structured interview/focus group schedule for dietetic team**

1. Has routine GS assessment changed your everyday practice or the number of patients referred to you?
2. Do the nurses communicate the reasons for referral to you?

If yes, was GS one of these reasons? How did they communicate it to you?

If not, how were the patients referred to you?

1. Did you use GS information in your assessment and treatment? If so, how did you use it?
2. Did you notice any differences in patients’ nutritional status between patients with normal GS levels and those with low GS level?
3. How did you manage patients with low GS levels? What were the interventions used? How did you record them?
4. How did you monitor patients with low GS levels during their stay in the hospital? How often did you see them? And how long did the interventions last?
5. Do you think using GS screening routinely at admission to hospital is useful or not?

**Semi-structure interview/focus group schedule for physiotherapy teams**

1. Has routine GS assessment changed your everyday practice or the number of patients referred to you?
2. Do the nurses communicate the reasons for referral to you?

If yes, was GS one of these reasons? How did they communicate it to you?

If not, how were patients been referred to you?

1. Did you use the GS information in your assessment and treatment? If so, how did you use it?
2. Did you notice any differences in patients’ physical function and ability to mobilise between patients with normal GS levels and those with low GS level?
3. What kind of physiotherapy activities did you use among patients with low GS levels? How practical was it for you to work with the patients with low GS? How practical was it for the patient with low GS to perform the exercises?
4. How did you monitor patients with low GS levels during their stay in the hospital? How often did you see them? And how long did the interventions last?
5. Do you think using GS screening routinely at admission to hospital is useful or not?

**Semi-structured interview schedule for patients**

I would like to obtain your experience and views about using the grip strength tester that has occurred few days ago

1. Did you understand the instructions given to you about how to use the grip tester?
2. Did you know what the grip test was testing?
3. Did you have any worries about the test before doing it? Can you describe your worry or anything in particular that concerned you about the procedure? Were your worries been addressed prior to the procedure?
4. Could you please describe to me what the grip test involved? Did you receive any encouragement during the test? How did you feel about that? Was there anything that could have been done to assure you and Help you during the procedure?
5. Where did the test take place? Were you sitting or lying on your bed during the test? Was the timing of the test convenient to you?
6. How did you find using the grip tester? Was it easy and comfortable or tiring and painful? was the time of the test agreed with you
7. Would you be prepared to perform the test again? Why?
8. What do you think of the idea that using grip test routinely to all patients admitted to hospital?
